# Active Microbial Communities Inhabit Sulphate-Methane Interphase in Deep Bedrock Fracture Fluids in Olkiluoto, Finland

**DOI:** 10.1155/2015/979530

**Published:** 2015-09-03

**Authors:** Malin Bomberg, Mari Nyyssönen, Petteri Pitkänen, Anne Lehtinen, Merja Itävaara

**Affiliations:** ^1^VTT Technical Research Centre of Finland, P.O. Box 1000, 02044 Espoo, Finland; ^2^Posiva Oy, Olkiluoto, 27160 Eurajoki, Finland

## Abstract

Active microbial communities of deep crystalline bedrock fracture water were investigated from seven different boreholes in Olkiluoto (Western Finland) using bacterial and archaeal 16S rRNA, *dsr*B, and *mcr*A gene transcript targeted 454 pyrosequencing. Over a depth range of 296–798 m below ground surface the microbial communities changed according to depth, salinity gradient, and sulphate and methane concentrations. The highest bacterial diversity was observed in the sulphate-methane mixing zone (SMMZ) at 250–350 m depth, whereas archaeal diversity was highest in the lowest boundaries of the SMMZ. Sulphide-oxidizing *ε*-proteobacteria (*Sulfurimonas* sp.) dominated in the SMMZ and *γ*-proteobacteria (*Pseudomonas* spp.) below the SMMZ. The active archaeal communities consisted mostly of ANME-2D and Thermoplasmatales groups, although Methermicoccaceae, Methanobacteriaceae, and Thermoplasmatales (SAGMEG, TMG) were more common at 415–559 m depth. Typical indicator microorganisms for sulphate-methane transition zones in marine sediments, such as ANME-1 archaea, *α*-, *β*- and *δ*-proteobacteria, JS1, Actinomycetes, Planctomycetes, Chloroflexi, and MBGB Crenarchaeota were detected at specific depths. *Dsr*B genes were most numerous and most actively transcribed in the SMMZ while the *mcr*A gene concentration was highest in the deep methane rich groundwater. Our results demonstrate that active and highly diverse but sparse and stratified microbial communities inhabit the Fennoscandian deep bedrock ecosystems.

## 1. Introduction

Stable deep terrestrial subsurface locations are presently being considered for long-term geological disposal of spent nuclear fuel. Microbe-mediated processes may play a key role in the long-term stability and risk assessments of such storage. Dissolved sulphide produced by sulphate reducing bacteria (SRB), for example, may exert influence on spent nuclear fuel canister corrosion leading to mobility of radionuclides [1]. In Olkiluoto, Finland, spent nuclear fuel will be disposed approximately 450 m deep in the bedrock. Therefore, understanding the role and functionality of microbial communities in this environment is of critical importance for the safety of the spent nuclear fuel repository [2].

Deep subsurface microbial communities of the Fennoscandian Shield, including Olkiluoto, are functionally diverse and play a role in a variety of redox reactions, such as nitrate, iron, and sulphate reduction, as well as methanogenesis (e.g., [3–6]). While the presence of these processes has been confirmed by cultivation based techniques [4, 5, 7] and DNA-based PCR techniques [3, 6, 8], activity of these processes* in situ* remains uncertain.

In general, deep subsurface microbial communities appear to have extraordinarily low metabolic activity [6]. However, under certain environmental conditions, such as sulphate-methane transition zones (SMTZ), microbial activity appears to increase dramatically [9, 10]. At SMTZs in marine sediments, concentrations of H_2_S increase (e.g., [11, 12]) possibly due to anaerobic oxidation of methane (AOM) and simultaneous reduction of SO_4_^2−^. In addition, both microbial cell concentration and microbial diversity have been seen to be elevated in sedimentary SMTZ environments [10]. Little is known of the activity, function, and composition of microbial communities in methane-rich deep terrestrial groundwater or terrestrial groundwater SMMZs.

Methane and sulphates are major constituents of Olkiluoto groundwater, residing in different groundwater layers [2]. Sulphate-rich water prevails at depths above 300 m below ground surface level (mbgsl) and methane-rich water dominates below 300 mbgsl. A sulphate-methane mixing zone (SMMZ) can be identified between 250 and 350 mbgsl [2]. In contrast to the clearly identifiable sharp SMTZs formed in anaerobic aquatic sediments [13, 14] the SMMZs in deep terrestrial groundwater are broad. In deep terrestrial subsurface, groundwater resides in bedrock fractures, which may be almost isolated and thereby exhibit stagnant groundwater or well connected with each other, which enables different degrees of groundwater flow. In addition, strong environmental changes, such as infiltration of surface water, crustal rebound, glaciation or deglaciation can affect the stability and position of the SMMZ [15].

Recently Pedersen et al. [16] simulated SMMZ mixing effect in Olkiluoto groundwater. By gradually increasing the concentration of sulphate in methane-rich and sulphate-poor groundwater over an experimental period of 103 days, the authors showed that the composition of the microbial community was strongly influenced by sulphate and methane. Several studies in Olkiluoto also show that the microbial communities in Olkiluoto groundwater are stratified and potentially affected by the groundwater SMMZ [3, 6, 17]. *δ*- and *γ*-proteobacteria are generally found in water layers above and in the SMMZ while *β*-proteobacteria become more abundant in the deeper methane-rich water [3, 6]. A clear increase in the number of methanogens was also detected simultaneously with a decrease in the number of sulphate reducing bacteria (SRB) in Olkiluoto deep groundwater [3, 17]. In addition, analysis of methyl coenzyme M reductase (*mcr*A) gene clone libraries demonstrates the presence of putative anaerobic methane oxidizing group 1 (ANME-1) archaea at 300–400 m depth [3].

Here, we extend this research and use RNA-targeted high-throughput (HTP) sequencing to investigate the active SRB and methanogen communities of the methane-rich deep groundwater around the depth of the nuclear waste repository rising up in to the SMMZ at the Olkiluoto site. In order to study the active microbial community in fracture water samples, the bacterial and archaeal 16S rRNA pools were also characterized and used as proxy for active (living) microbial cells. In addition, the abundance of SRB and methanogen communities was studied by qPCR targeting dissimilatory sulfite reductase (*dsr*B) and* mcr*A transcripts and genes.

## 2. Materials and Methods

### 2.1. Description of the Site

The island of Olkiluoto is the selected site for deep (approximately 450 mbgsl) geological disposal of spent nuclear fuel in Finland. The island has almost 60 boreholes drilled for research and monitoring purposes and studies on the chemistry and microbiology of the groundwater have been on-going since the 1980s [2]. The groundwater in Olkiluoto is stratified relative to physicochemical parameters [18]. From the surface to a depth of 30 mbgsl the water is of meteoric origin (i.e. precipitation) and the water type is fresh to brackish. The uppermost 100 mbgsl has a high concentration of dissolved inorganic carbon (as bicarbonates), and salinity (as total dissolved solids [TDS] and chlorine) increases with depth. Between 100 and 300 mbgsl, salinity is roughly similar to the present day Baltic Sea, but, below 300 mbgsl, the salinity increases up to 84 g TDS L^−1^ at 1000 mbgsl. Based on drill core logging, the bedrock of Olkiluoto consists mainly of gneiss (9% of the bedrock volume), migmatitic gneiss (64% of the bedrock volume), TGG (tonalite-granodiorite-granite) gneiss (8%), and pegmatitic granite (19%) [19]. In addition, of the migmatitic gneiss 67% is veined and 33% diatexitic gneiss.

Between 100 and 300 mbgsl, the SO_4_^2−^ concentration is elevated in ancient (i.e., pre-Baltic) seawater derived groundwater. Below this layer, the methane concentration in the water increases and Cl^−^ dominates whereas SO_4_^2−^ is almost absent. A mixing zone where methane-rich groundwater diffuses into sulphate-rich groundwater (a sulphate-methane mixing zone, SMMZ) can be identified at 250 to 350 mbgsl depth. This zone is characterized by increased concentration of sulphide and a decrease in sulphate and methane.

The temperature rises linearly with depth, from ca. 5-6°C at 50 mbgsl to ca. 20°C at 1000 mbgsl [20]. The pH of the water is slightly alkaline throughout the depth profile. Several aquifer zones, such as zones HZ20 or HZ21, span several different boreholes (Table 1).

### 2.2. Sampling

**Figure 1:**
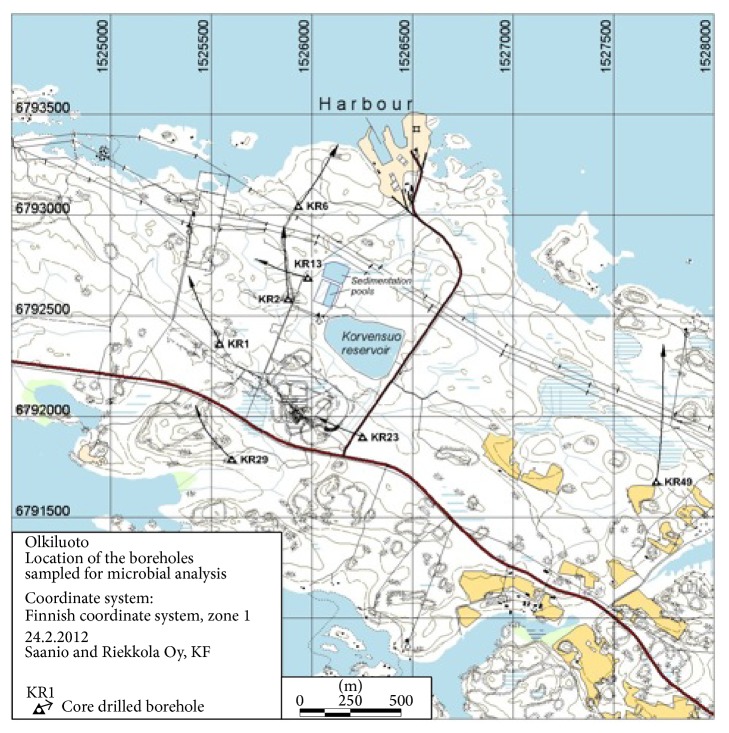
Map of Olkiluoto area where the different boreholes sampled in this study are indicated as open triangles. The arrows show the direction in which the boreholes lead. The scale bar is equal to 500 m.

Deep groundwater samples (Table 1) from specific fracture zones were collected from seven different boreholes in Olkiluoto (Figure 1) between December 2009 and May 2010. Fracture zones were isolated by permanent or temporary inflatable packers as described previously [3]. Packer-sealed fracture zones were purged by pumping for at least four weeks prior to sampling in order to allow indigenous fracture water to fill the isolated borehole section. Anaerobic groundwater was pumped from the borehole directly in to an anaerobic chamber (MBRAUN, Germany) through a sterile, gas-tight polyacetate tube (8 mm outer diameter), where samples were collected in acid-washed, sterile 2 L Schott glass bottles (Duran Group GmBH, Germany). Microbial biomass for nucleic acid analyses was concentrated from 500 mL and 1000 mL samples by vacuum filtration through cellulose acetate membranes (0.2 *µ*m pore size, Corning, MA, USA) inside the glove box. Filters were then cut out from the filter funnels and frozen on dry ice in sterile 50 mL cone tubes (Corning MA, USA). Frozen samples were transported on dry ice to the laboratory where they were stored at −80°C prior to analysis.

Samples for microbial cell counts were collected in acid-washed sterile, anaerobic 100 mL glass infusion flasks equipped with butyl rubber septa and aluminium crimp caps and transported to the laboratory at 4°C in a light-proof container. The samples were analysed within 2 days of sampling.

### 2.3. Geochemistry

The geochemical data were provided by Posiva Oy and are presented in Table 1. Measurements were performed as described in Table 2.

### 2.4. Total Cell Counts

The total number of cells (TNC) was determined by fluorescent staining with 4′,6-diamidino-2-phenylindole (DAPI) [21] with slight modifications. A 5 mL subsample of each groundwater sample was stained with DAPI (1 *µ*g mL^−1^) for 20 min at room temperature in the dark and collected on black polycarbonate Isopore Membrane filters (0.2 *µ*m GTBP, Millipore, Ireland) with the Millipore 1225 Sampling Manifold (Millipore, USA) under low vacuum. The filters were rinsed with 1 mL filter sterilized 0.9% NaCl prior to and after filtration. Fluorescent cells were visualized under UV light with an epifluorescence microscope (Olympus BX60, Olympus Optical Ltd., Tokyo, Japan) and 1000x magnification. The number of cells was calculated from 30 random microscopy fields according to the magnification factor, filtered volume, and the surface area of the filter used [22].

### 2.5. Nucleic Acid Isolation

Microbial community nucleic acids (DNA and RNA) were isolated directly from the frozen cellulose-acetate filters with the PowerSoil DNA or PowerWater RNA extraction kit (MoBio Laboratories, Inc., Solana Beach, CA), respectively. Filters for DNA extraction were cut into 2 × 2 mm pieces with sterile scalpels in a laminar flow hood before insertion into the lysis tube. Nucleic acids were isolated according to the manufacturer's instructions except that for DNA extraction, the microbial cells were lysed by bead beating with a Precellys (Bertin Technologies, France) homogenizer for 30 s with 5 s increments at room temperature. The DNA and RNA from 500 mL and 1000 mL samples were eluted in 50 *µ*L elution buffer and 100 *µ*L elution buffer, respectively. Three replicate filters were used for DNA or RNA isolation. Negative isolation controls were performed from clean cellulose-acetate filter units in parallel with the samples using the same protocol and reagents as for the samples.

Residual DNA in the RNA extracts was checked by PCR with the primers used in this study (Table 3). If no PCR product was obtained, it was assumed that all residual DNA was successfully removed and the RNA extract was submitted to cDNA synthesis. If a PCR product was obtained, the RNA extract was treated with DNase (Promega, WI, USA) according to the manufacturer's instructions. cDNA was synthesized by first incubating 11.5 *µ*L aliquots of RNA extract together with 250 ng random hexamers (Promega, WI, USA) and 0.83 mM final concentration dNTP (Finnzymes, Espoo, Finland) at 65°C for 5 minutes before cooling the reactions on ice for 1 minute. The reverse transcription was then performed with the Superscript III kit (Invitrogen), by adding 4 *µ*L 5x First strand buffer, 40 U DTT, and 200 U Superscript III to the cooled reactions. To protect the RNA from degradation, 40 U of recombinant RNase inhibitor, RNaseOut (Promega, WI, USA), was used. The reactions were incubated at 25°C for 5 minutes, 50°C for 1 h, and 70°C for 15 min. Three parallel reactions were performed for each sample as well as for the reagent controls. The parallel reactions were subsequently pooled.

### 2.6. Amplicon Library Preparation

Libraries for 454 high-throughput (HTP) amplicon sequencing were prepared by PCR from the cDNA samples. Bacterial 16S rRNA fragments covering the V1–V3 variable regions were amplified with primers 8F and P2 equipped with adapter and MID sequences at their 5′ end in a single round PCR (Table 3) [23, 24]. Archaeal 16S rRNA fragments were produced with a nested PCR using primers A109f and Arch915R [25, 26] for the first round and tagged primers ARC344f and Ar744r [27, 28] covering the V3-V4 variable areas for the second round.* Dsr*B fragments were amplified in a single round PCR with tagged primers 2060F [29] and* dsr*4R [30].* Mcr*A fragments were obtained by nested PCR. Initially, a 1.2 kb* mcr*A fragment was amplified with primers* mcr*A412f and* mcr*1615r [3]. The product of this PCR was then amplified with tagged primers ME1 and ME3r modified from [31]. PCRs were performed with Phusion DNA polymerase (Finnzymes, Espoo, Finland) in 1x HF buffer. Each 50 *µ*L reaction contained 0.5 mM dNTP and 1 *µ*M of primers. The PCR conditions consisted of an initial denaturation step of 30 s at 98°C, followed by 35 cycles of 10 s at 98°C, 15 s at 55°C, 15 s at 72°C, and a final extension step at 72°C for 5 min. Two replicate samples were used for each borehole depth and a minimum of two amplification reactions were performed for each replicate sample, which were subsequently pooled prior to sequencing. All PCR reactions were also run with the negative nucleic acid extraction and reagent controls. The sequencing was performed at the Institute of Biotechnology, University of Helsinki, Finland, using the FLX 454 (454 Life Sciences, Branford, CT, USA).

### 2.7. Real-Time Quantitative PCR

The abundance of bacterial* dsr*B and archaeal* mcr*A genes and transcripts was determined by qPCR with KAPA SYBR Fast 2x Master mix for Roche LightCycler 480 (Kapa Biosystems, Inc., Boston, MA, USA). Reactions were performed in triplicate for each sample. Each reaction contained 1 *µ*L of extracted DNA or cDNA as template and 5 pmol of both forward and reverse primers (Table 3). The qPCR was performed on a Roche LightCycler 480 (Roche Applied Science, Germany) on white 96-well plates (Roche Applied Science, Germany) sealed with transparent adhesive seals (4titude, UK). The qPCR conditions consisted of an initial denaturation at 95°C for 10 minutes followed by 45 amplification cycles of 15 seconds at 95°C, 30 seconds at 55°C, and 30 seconds at 72°C with a quantification measurement at the end of each elongation. A final extension step of three minutes at 72°C was performed prior to a melting curve analysis. The melting curve analysis consisted of a denaturation step for 10 seconds at 95°C followed by an annealing step at 65°C for one minute prior to a gradual temperature rise to 95°C at a rate of 0.11°C s^−1^ during which the fluorescence was continuously measured. The number of gene and transcript copies was calculated by comparing the amplification result (Cp) to that of a dilution series of plasmids containing* mcr*A or* dsr*B genes ranging from 0 to 10^7^ gene copies per reaction as described in Nyyssönen et al. [3]. The lowest detectable standard concentration for the* dsr*B qPCR was 16* dsr*B gene copies/reaction. In the* mcr*A qPCR assay, the lowest detectable standard had 100* mcr*A copies/reaction. Template inhibition of the qPCR was tested by adding 2.17 × 10^4^ plasmid copies containing fragment of the morphine-specific Fab gene from* Mus musculus* gene to reactions containing template DNA or cDNA and comparing the result to a dilution series of the plasmid as described in [3]. The inhibition of the qPCR assay by the template DNA was found to be low. The average Crossing point (Cp) value for the standard sample (2.17 × 10^4^ copies) was 28.7 (±0.4 std), while for the DNA samples the Cp was 28.65–28.91 (±0.03–0.28 std) and for the cDNA samples was 28.69–28.96 (±0.02–0.23 std). Nucleic acid extraction and reagent controls were run in all qPCRs in parallel with the samples. Amplification in these controls was never higher than the background obtained from the no template controls.

### 2.8. Sequence Processing and Analysis

Sequence reads were trimmed with Mothur (v 1.31.2) [32] to remove adapter, barcode, and primer sequences and to exclude sequences that did not meet the quality criteria (i.e., no barcode and primer mismatches, no ambiguous nucleotides, maximum eight nucleotide long homopolymer stretches, and defined minimum length). The minimum length was 300 bp for bacterial 16S rRNA and* dsr*B sequences and 200 bp for archaeal 16S rRNA and* mcr*A sequences. The bacterial and archaeal 16S rRNA sequences were aligned with Mothur [32] using a Silva reference alignment [33] for bacterial (14 956 sequences) and archaeal (2 297 sequences) 16S rRNA gene sequences, respectively. The* dsr*B sequences were aligned with Geneious Pro (v 5.6, Biomatters Ltd., New Zealand) using a* dsr*AB model alignment [34] (97 sequences). The* mcr*A sequences were aligned with Mothur using a* mcr*A gene sequence model alignment (this study) (213 sequences). The alignments from the amplicon libraries were checked and manually corrected with Geneious Pro before further analysis with Mothur.

The sequences were divided into operational taxonomic units (OTUs) based on 97% sequence homology for the bacterial and archaeal 16S rRNA sequences and the* dsr*B sequences and 99% for the* mcr*A sequences. The sequencing coverage was evaluated by rarefaction analysis and the estimated species richness and diversity indices were calculated in Mothur.

The bacterial and archaeal 16S rRNA sequences were taxonomically classified with Mothur using the GreenGenes 13_8 database [35]. The representative sequences of the* dsr*B and* mcr*A OTUs were analysed using the Geneious Pro (Biomatters Inc., New Zealand). The* dsr*B and* mcr*A sequences were imported into Geneious Pro and aligned to reference sequences and most closely matching sequences determined against the NCBI database with blastn tool in Geneious Pro. The alignments were performed with Muscle [36] using default settings and the alignments were edited manually. The* mcr*A and* dsr*B sequences were subsequently translated to amino acid sequences before phylogenetic analyses. Phylogenetic analyses were performed on the alignments using PhyML [37] with the Jukes-Cantor (JC69) [38] substitution model for nucleic acid sequences and the Whelan-Goldman substitution model [39] for amino acid sequences. Bootstrap support for nodes was calculated based on 1000 random repeats.

For comparable *α*- and *β*-diversity analyses the data sets were normalized by random subsampling according to the sample with the lowest number of sequence reads, that is, 1200, 893, 2249, and 2324 sequences for archaea, bacteria,* dsr*B, and* mcr*A, respectively.

The sequences have been submitted to the European Nucleotide Archive (ENA, https://www.ebi.ac.uk/ena/) under accession numbers ERS514153–ERS514176.

### 2.9. Statistical Analyses

Statistical analyses were calculated with PAST v. 3.0 [40] in order to determine which of these parameters correlated most strongly with the detected taxa. The Shapiro-Wilk test [41] and Anderson-Darling test [42] were performed to analyze the normal distribution of the geochemical parameters. For sample parameters with *P* < 0.05 normal distribution were rejected and these parameters were excluded from the correlation calculations. The excluded parameters were DIC, bicarbonate, alkalinity, sulphate, S_tot_, N_tot_, Fe(II), F_tot_, Sr, 16S rRNA gene copies mL^−1^, and* dsr*B transcripts mL^−1^ and* mcr*A genes mL^−1^. Pearson's linear *r* correlation between presence and absence of different taxa in correlation to the geochemical parameters was calculated with PAST.

## 3. Results and Discussion

The crystalline bedrock of Olkiluoto has been chosen to host the deep geological repository for spent nuclear fuel in Finland. The spent nuclear fuel will be stored in copper canisters with nodular cast iron insert at 450 m depth and isolated from the bedrock by bentonite clay. Groundwater salinity and carbon content at different depths as well as the increase in the amount of CH_4_ and H_2_S and decrease in the amount of SO_4_^2−^ at specific depths suggest the existence of a broad sulphate-methane mixing zone (SMMZ) in the groundwater at approximately 250–350 mbgsl depth [2]. At corresponding sulphate-methane transition zones (SMTZ) in marine sediments both the microbial activity and the diversity of the microbial communities increase dramatically [9, 43]. If the same kind of intensified activity occurs in groundwater SMMZs an increased risk may arise for, for example, microbially induced sulphate reduction aided corrosion of the waste capsules, release of radioactive waste, and mobilization of radionuclides.

In this study, we investigated the transcriptionally active microbial communities of the deep methane-rich groundwater spanning the depth of the future spent nuclear fuel repository. Triplicate groundwater samples from depths between 296 and 798 mbgsl from seven different boreholes in Olkiluoto were collected in order to characterize the active microbial communities around the depth of the planned repository (Table 1, Figure 1). The samples represented brackish SO_4_^2−^ rich water and saline methane-rich water (as classified in [2]). The carbonate content in the groundwater generally decreased with depth whereas in deeper water the concentration of methane increased from almost none at 296 m to more than 900 mL L^−1^ gas at 800 mbgsl. The concentration of SO_4_^2−^ was highest (379 mg L^−1^ groundwater) in the sample from 328 mbgsl and decreased radically with depth. The H_2_S concentration was also highest at 296–347 mbgsl and decreased with depth.

**Figure 2:**
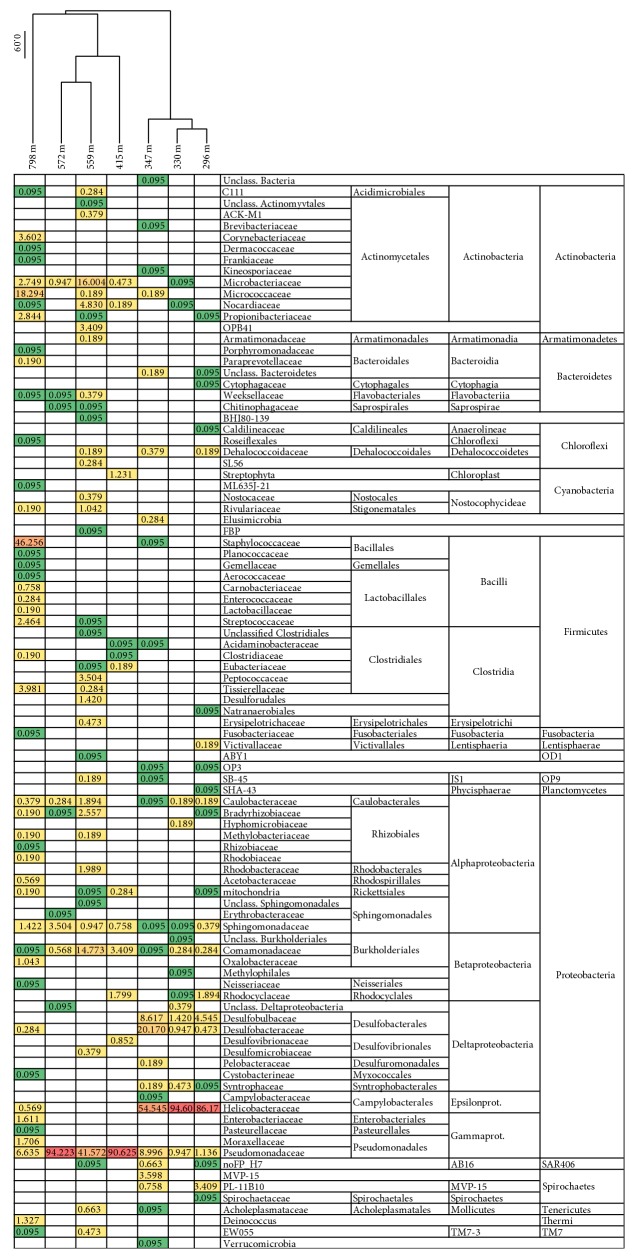
The relative distribution of bacterial 16S rRNA sequence reads belonging to specific bacterial families. The relative abundance of sequence reads are highlighted by color, where green represents the lowest relative abundance, yellow represents medium abundance, and red represents high abundance. The samples were clustered using the Morisita-Horn algorithm in Mothur. The data were normalized between the different samples to include 893 random sequence reads from each sample.

**Figure 3:**
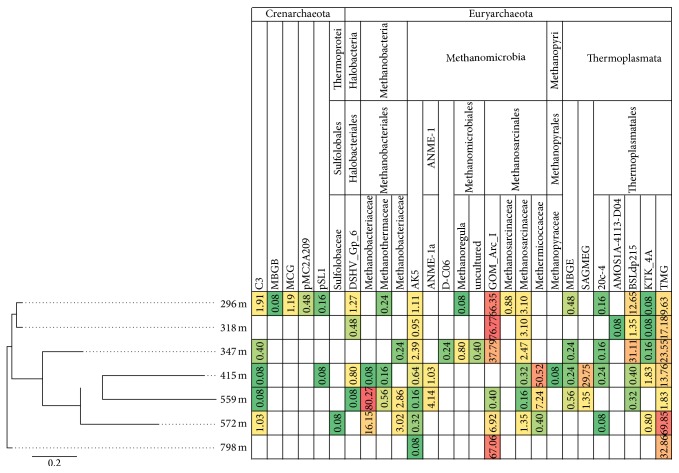
The relative distribution of archaeal 16S rRNA sequence reads belonging to specific archaeal families. The relative abundances of sequence reads are highlighted as described in Figure 2. The samples were clustered as described in Figure 2. The data was normalized between the different samples to include 1200 random sequence reads from each sample.

**Figure 4:**
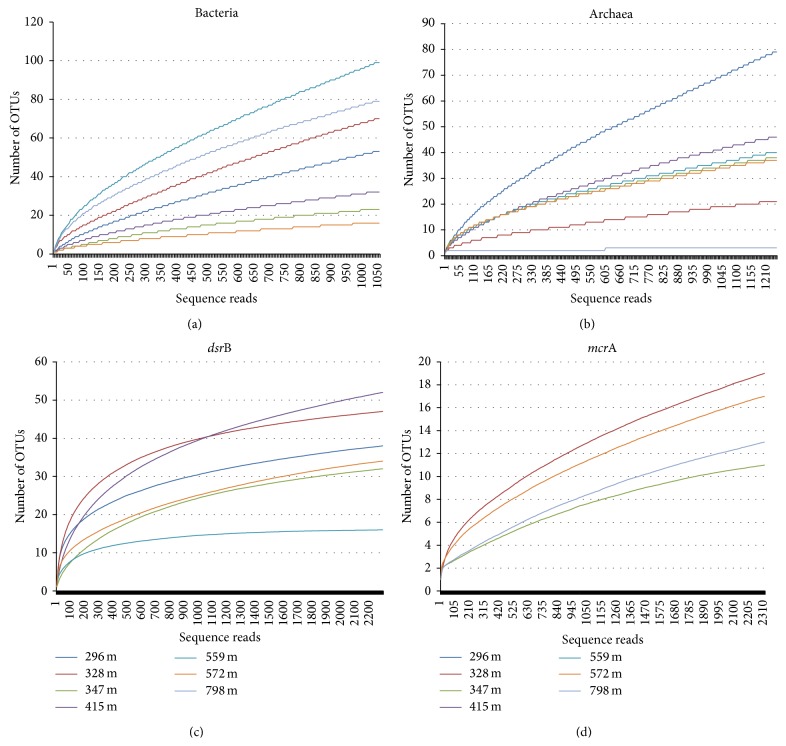
Rarefaction curves of the sequence data obtained from each RNA extract normalized to equal number of sequence reads per sample: (a) bacterial 16S rRNA, (b) archaeal 16S rRNA, (c)* dsr*B transcripts, and (d)* mcr*A transcripts. The *x*-axis displays the number of sequence reads and the *y*-axis displays the number of different OTUs obtained. Figures (a)–(c) present rarefaction values at the distance 0.03 and (d) rarefaction for distance 0.01.

The TNC mL^−1^ groundwater varied between 4.2 × 10^5^ mL^−1^ at 296 m and 1.5 × 10^4^ mL^−1^ at 415 mbgsl with a general decline with depth (Table 1). HTP sequencing of bacterial and archaeal 16S rRNA with 454 technologies identified a total of 95 bacterial families and 27 archaeal families in the seven analyzed samples (Figures 2 and 3). The rarefaction analyses showed that the bacterial and archaeal communities were well characterized from 415 to 572 mbgsl (Figure 4). In the remaining samples, between 16 and 52% of the estimated bacterial and archaeal OTU richness was captured by sequencing.

**Figure 5:**
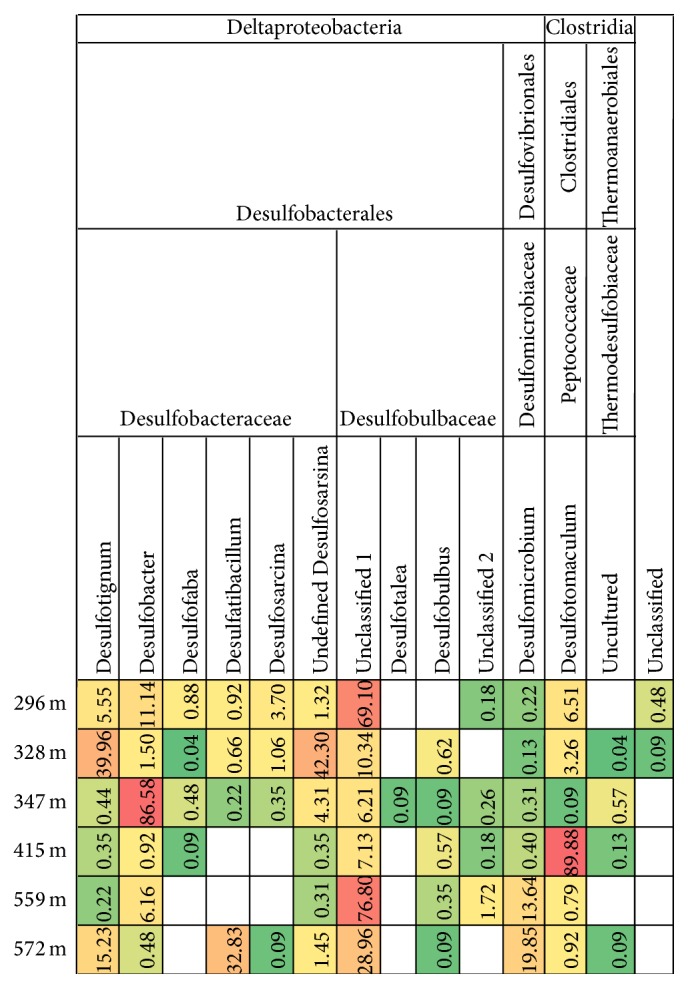
The relative distribution of* dsr*B transcript sequence reads belonging to specific SRB families according to the phylogenetic identification of the sequences presented in Figure 6. The relative abundance of sequence reads and the clustering of the samples are presented as described in Figure 2. The data were normalized between the different samples to include 2249 random sequence reads from each sample.

**Figure 6:**
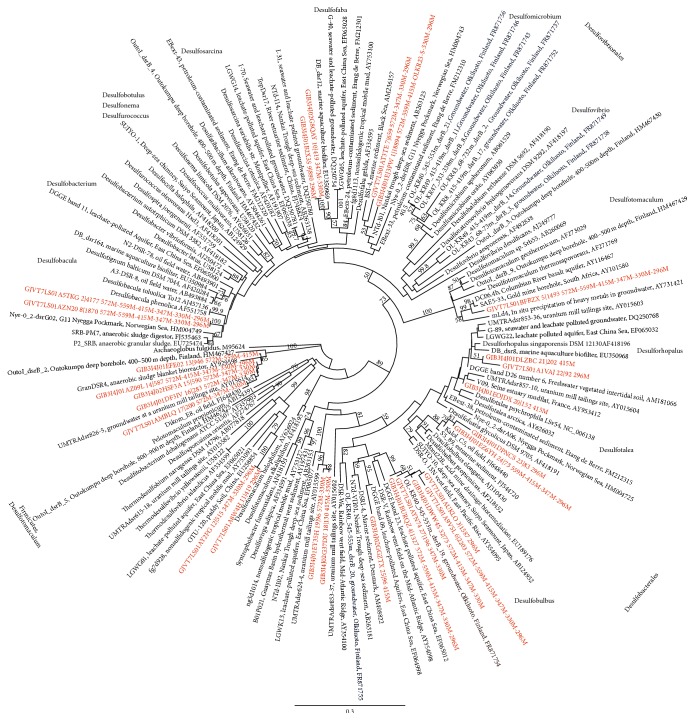
The phylogenetic distribution of the amino acid sequences of the OTUs of* dsr*B transcripts detected in this study presented as a maximum likelihood tree. The sequences detected in this study are shown in red. Bootstrap support for nodes was calculated with 1000 random repeats and nodes with more than 50% support are indicated. Sequences detected in this study are shown in red. The sequence name codes consist of the sequence IDENTIFIER and the OTU number|the number of sequence reads in that OTU followed by the depths from which this OTU has been detected.

**Figure 7:**
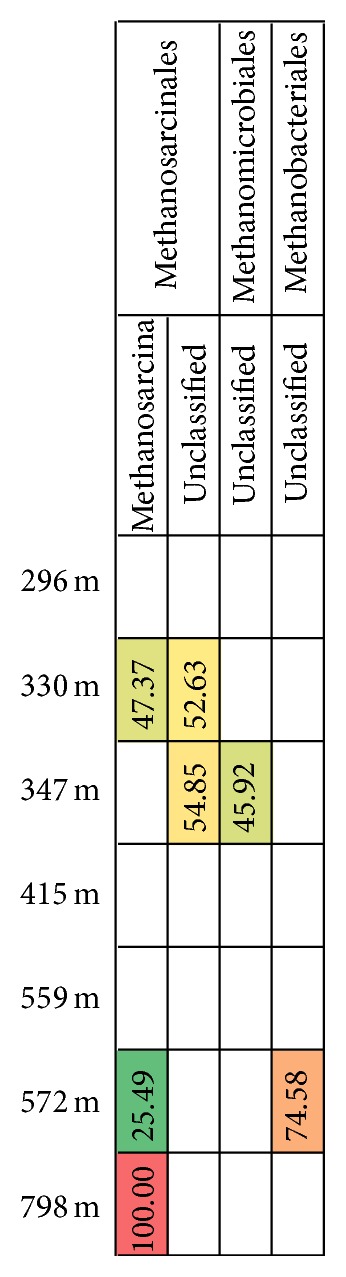
The relative distribution of* mcr*A transcript sequence reads belonging to specific methanogenic archaeal families based on the phylogenetic identification of the* mcr*A reads as presented in Figure 8. The relative abundances of sequence reads are highlighted as described in Figure 2. The data was normalized between the different samples to include 2324 random sequence reads from each sample.

**Figure 8:**
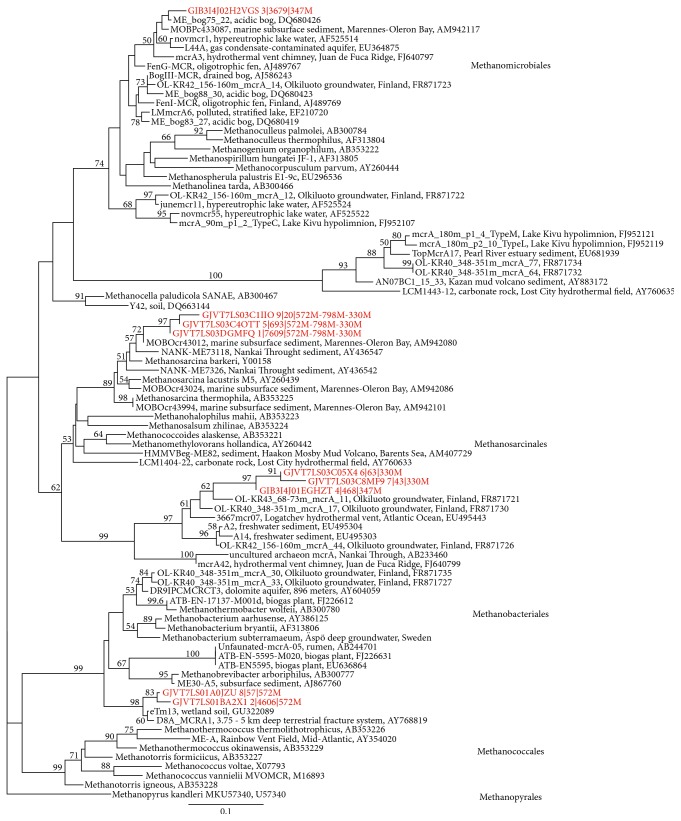
The phylogenetic distribution of the amino acid sequences of the OTUs of* mcr*A transcripts obtained detected in this study presented as a maximum likelihood tree. The sequences detected in this study are shown in red. Bootstrap support for nodes was calculated with 1000 random repeats and nodes with more than 50% support are indicated. The sequence codes are as described in Figure 6.

*dsr*B gene transcripts were obtained from sequencing from depths between 296 mbgsl and 572 mbgsl, but not from the deepest sample from 798 mbgsl. The* dsr*B sequences belonged to six different SRB families and 14 genera (Figures 5 and 6). The* dsr*B transcript diversity was well covered showing between 81 and 98% of the estimated Chao1 OTU richness obtained. Transcripts of the* mcr*A genes were obtained for 454 sequencing with nested PCR amplification from four different depths, 328 m, 347 m, 572 m, and 798 mbgsl (Figure 7). The* mcr*A transcripts belonged to four methanogenic genera (Figure 8) that covered the Chao1 estimation of the total* mcr*A diversity.

Diversity of the active microbial communities was highest at sampling depths between 296 and 347 mbgsl, that is, in the SMMZ. At this depth, both bacterial diversity (*H*′ = 1.8, normalized to equal number of sequence reads/sample) and SRB (*H*′ = 2.29 and 2.65) diversity were the highest (Table 4). The highest archaeal diversity (*H*′ = 1.91), in contrast, was seen in the lowest boundaries of the SMMZ at 347 mbgsl. The diversity of the methanogenic communities was low in all samples from which sequences were obtained by nested PCR (*H*′ = 0.42–0.76).

### 3.1. Sulphate-Methane Mixing Zone (SMMZ)

The structure of the active bacterial communities was similar between samples derived from similar depth of the different boreholes but changed with greater depth intervals (Figure 2). Sampling depths between 296 and 347 mbgsl contain the most H_2_S and SO_4_^2−^ rich water in this study and are influenced by a fraction of the methane-rich groundwater from deeper groundwater layers. Here, the most abundant bacterial group was *ε*-proteobacteria of the Helicobacteraceae family mostly belonging to the* Sulfurimonas*. This group formed 54–95% of the active bacterial communities as determined by the total number of sequences. *ε*-proteobacteria are believed to be enriched in the vicinity of SMTZs in marine sediments [44] and many are mesophilic, H_2_- and sulphur-oxidizing chemolithoautotrophs [44–46]. They may play a profound role in recycling H_2_S to SO_4_^2−^ and are also a significant group in SMMZ microbial communities [10] where they fix CO_2_ at the expense of sulphides and other electron donors. By fixing CO_2_, they may account for a significant amount of assimilated carbon compounds available to microbial communities in deep subsurface environments [47]. The second largest group at 296–347 mbgsl was Desulfobacterales *δ*-proteobacteria forming 2–29% of the active community based on 16S rRNA (Figure 2). This is in accordance with the detection of the* dsr*B gene transcripts similar to uncultured group 1 Desulfobulbaceae of the Desulfobacterales family at this depth. These* dsr*B transcripts formed more than 69% of the* dsr*B transcripts at 296 mbgsl and showed a positive and significant correlation (>0.8, *P* < 0.01) with pH between 7.9 and 8.1. At 328 mbgsl,* dsr*B transcripts of the genera* Desulfotignum* and undefined* Desulfosarcina* of the Desulfobacteraceae were the most common. The amount of* dsr*B genes varied between 0.5 and 3.1 × 10^4^ copies mL^−1^ at 296–374 mbgsl. In addition, the highest transcriptional activity of the* dsr*B genes, 1.2–2.9 × 10^2^ transcripts mL^−1^, was detected here, coinciding with the highest sulphate and sulphide concentrations and the lowest methane concentrations measured in this study.

At 296–347 mbgsl, a minor portion of the bacterial community belonged to methylotrophic *β*-proteobacteria and Verrucomicrobia, which may be capable of methane oxidation in the SMMZ (Figure 2). However, a more likely scenario for methane oxidation is the AOM process performed by archaeal ANME linages. Nyyssönen et al. [3] reported putative ANME-1* mcr*A genes from the 300 to 400 mbgsl in Olkiluoto. In the present study, the active archaeal communities detected in the SMMZ mainly consisted of GOM_Arc_I Methanosarcinales (Figure 3), which also are known as the ANME-2D. ANME-2D archaea have been shown to independently perform nitrate mediated AOM without the need for a bacterial partner [48]. This is in agreement with the* mcr*A gene transcripts detected at this depth, which mostly (55–100%) belonged to Methanosarcinales groups.

At the lower boundaries of the SMMZ at 347 mbgs, the active SRB community changed and the* dsr*B gene transcript pool was dominated by transcripts belonging to an uncultured Desulfobacteraceae group of SRB most closely related to* Desulfobacter* (86.5%), overlapping the distribution of ANME-1 in Olkiluoto.* Desulfosarcina dsr*B transcripts were found only at low abundance but were most numerous at 296–328 mbgsl. Together with the* Desulfobacter* the* Desulfosarcina* also belongs to the Desulfobacteraceae. These* Desulfosarcina* have been reported to form AOM consortia with ANME-1 and ANME-2 archaea [49], which may indicate that these associations also occur in Olkiluoto groundwater SMMZ.

### 3.2. Methane-Rich Groundwater

Below the SMMZ, at 415–572 mbgsl, the sulphate concentration in the groundwater is greatly reduced, the groundwater salinity increased, and the methane concentration is high. At this depth, *γ*-proteobacteria most similar to* Pseudomonas* species dominated (41–94%) the active bacterial communities. These bacteria may be the major CO_2_-fixing bacteria in Olkiluoto deep methane rich groundwater, as they have been shown to be in the Baltic Sea [50].

A peak in the bacterial diversity was seen at 559 mbgsl in the methane-rich groundwater. Several SMTZ signature groups were detected at this depth including putatively methylotrophic *α*- and *γ*-proteobacteria, *β*-proteobacteria, *δ*-proteobacterial SRB, JS1, Actinomycetes, Planctomycetes, and Chloroflexi. *β*-proteobacteria belonging to the Burkholderiales, for example, are believed to be the sole bacterial partner performing nitrification in the AOM association with ANME-2c archaea [51]. *β*-proteobacterial families Sphingomonadaceae and Comamonadaceae were detected as minority (<3.5%) at all depths. *β*-proteobacteria were a major group only at 559 mbgsl where* Acidovorax* sp. (Comamonadaceae) contributed almost 15% of the active community and correlated positively and significantly with the highest pH measured in the present study. A low abundance (<1%) of methanotrophic *α*-proteobacterial Methylobacteriaceae (*Methylobacter* sp. and* Methylocystis* sp.) correlating significantly with depth and salinity were found at this depth. Similar methanotrophs have readily been isolated from anaerobic methane-rich deep subsurface environments, such as terrestrial mud volcanoes [52]. Wrede et al. [52] suggested that aerobic methane oxidation could be activated whenever oxygen was available and thereby keep the subsurface ecosystem anaerobic.

Methylotrophic Methermicoccaceae and SAGMEG Thermoplasmata were the most abundant archaea at 415 mbgsl (50.5% and 29.8%, resp.). Hydrogenotrophic Methanobacteriaceae, which correlated with the highest pH, were the most abundant archaea at 559 mbgsl (80.3%) and terrestrial miscellaneous group (TMG) Thermoplasmatales at 572 mbgsl (69.9%). Nevertheless, the* mcr*A transcripts at 572 mbgsl mostly (75%) belonged to Methanobacteriales methanogens. ANME-1 archaea were found in the methane-rich groundwater at 415 and 559 mbgsl (4.1% and 1.0%, resp.) and correlated positively although not significantly with the highest pH values measured in this study. ANME-1 archaea were most abundant at depths where the GoM_Arch_I/ANME-2D archaea were mainly absent. Recent research shows that some ANME groups are capable of performing sulphate mediated AOM on their own [53], where they form S_2_ by a so far unknown sulphate reduction process. The SO_4_^2−^-mediated AOM performed by the ANME-1 could dominate specifically at 415–559 mbgsl, where the concentration of methane increases dramatically. Our results are similar to those of Pedersen [54], who suggested that a sulphate mediated AOM process coupled to sulphate reduction may occur in Olkiluoto groundwater at the SMMZ depth, although they did not obtain conclusive evidence for this process.

In the methane-rich water, the methanogens and SRB were clearly enriched at different depths. At 559 mbgsl where the highest number of* mcr*A genes (4.6 × 10^2^ copies mL^−1^) was detected the number of* dsr*B genes was only 6.5 × 10^1^ copies mL^−1^ and no* dsr*B transcripts could be detected by qPCR.* Dsr*B genes in contrast were abundant above (1.6 × 10^4^ copies mL^−1^ at 415 mbgsl) and below (2.2 × 10^3^ *dsr*B copies mL^−1^ at 572 mbgsl) this depth although the sulphate concentration in the water was only 0.5–1.4 mg L^−1^. The reason for the higher amount of* dsr*B gene copies mL^−1^ in the sulphate poor water may be that the SRB live by fermentation instead of sulphate reduction. For example,* Desulfobulbus* and* Desulfotomaculum* species have been shown to reduce Fe(III) during fermentation of pyruvate [55, 56]. Both of these sulphate reducers were abundant in the methane rich and sulphate poor groundwater. At 415 mbgsl* Desulfotomaculum dsr*B gene transcripts were the most abundant (89.9%) while Desulfobulbaceae family 1 of the Desulfobacterales dominated (76%) at 559 mbgsl and showed positive and significant correlation with pH the highest groundwater pH. The most even distribution of* dsr*B gene transcripts was seen at 572 mbgsl, where* Desulfatibacillum* (>32%),* Desulfomicrobium* (>19%), and uncultured Desulfobulbaceae (uncultured 1) (>28%) dominated the SRB communities. Firmicutes* dsr*B gene transcripts other than those belonging to* Desulfotomaculum* were detected only at <1% relative abundance at 328 mbgsl and were present at 296–415 mbgsl and 572 mbgsl (Figure 6). These* dsr*B sequences all belonged to* Thermodesulfovibrio* species previously found in soil environments.

### 3.3. Deep Methane-Rich Groundwater

At 798 mbgsl, the groundwater is highly saline with over 53 g dissolved solids L^−1^ and a high concentration of methane. The microbial community at this depth was clearly different from those at the other depths. However, the bacterial diversity at this depth was surprisingly high. The most common bacterial groups were the Bacillales and Actinobacteria, which significantly increased with increasing depth and salinity throughout the studied depth profile and formed 46% and 18% of the active bacterial community. Archaeal diversity was low, and the archaeal community consisted mainly of GOM_Arc_I Methanosarcinales/ANME-2D (67%) and TMG archaea (32.9%) (Figure 3). No* dsr*B or* mcr*A genes or transcripts were detected by qPCR despite the relatively high microbial density, 2.3 × 10^4^ cells mL^−1^. In accordance, no* dsr*B transcripts were obtained for 454 sequencing either.* mcr*A transcripts were obtained by the nested PCR approach only, and all sequences belonged to Methanosarcinales methanogens. The coappearance of these* mcr*A transcripts together with the high relative abundance of GOM_Arc_I Methanosarcinales/ANME-2D archaea indicates active methane cycling activity of GOM_Arc_I Methanosarcinales/ANME-2D archaea at this depth.

## 4. Conclusions

We observed a clear change in the active microbial community composition at the sulphate-methane interface and the methane-rich groundwater in Olkiluoto. Several SMTZ signature groups were detected, as well as a high diversity of active microorganisms. We found a characteristic increase in the transcription of the* dsr*B gene in the sulphate reducing and putative AOM zone between 296 and 347 mbgsl, coinciding with* mcr*A transcripts of methylotrophic methanogens that possibly belong to the ANME-2D. In methane-rich water between 415 m and 559 mbgsl the ANME-2D were few or absent, while ANME-1 archaea appeared.* mcr*A transcripts from an uncultured group of Methanosarcinales archaea cooccurred with the ANME-2D archaea, but whether they produce or oxidize methane using the reverse methanogenesis pathway is not known.

Overall the active microbial communities in Olkiluoto deep groundwater are diverse and SRB and methanogens are not the only microbial groups to have an influence on hydrogeochemical conditions and to further be taken into account in the safety case of the disposal of spent nuclear fuel. AOM may also be mediated by means other than sulphate or nitrate reduction by different bacterial groups. The great abundance of bacterial and archaeal taxa generally not involved in methane production or oxidation, or nitrate or sulphate reduction, also indicate that the main energy converting metabolic pathways may, in the absence of oxygen, be fermentation of organic molecules.

## Figures and Tables

**Table 1:** The geochemical and biological measurements from the samples collected from fracture fluids from seven different boreholes in Olkiluoto, Finland. The different boreholes are presented as sampling depths.

	296 m	328 m	347 m	415 m	559 m	572 m	798 m
Borehole	OL-KR13	OL-KR6	OL-KR23	OL-KR49	OL-KR2	OL-KR1	OL-KR29
Depth below ground surface (m)	−296.11	−328.37	−346.52	−415.45	−559.15	−572.24	−797.81
Water type	Brackish SO_4_	Brackish SO_4_	Saline	Saline	Saline	Saline	Saline
Transmissivity (m^2^ s^−1^)	5.86 × 10^−8^	1.31 × 10^−7^	6.48 × 10^−7^	4.37 × 10^−7^	4.33 × 10^−7^	5.50 × 10^−7^	(<10^−9^)
Hydrogeological zone	HZ001		HZ20A		HZ21	HZ21	
Pump rate (mL min^−1^)	22	104	20	172	23.9	62.1	6.1
Cumulative volume fracture fluid removed (L)	1129	5486	971	7509	1251	4492	496
Sampling date Microbiology	9.3.2010	18.5.2010	15.12.2009	14.12.2010	27.1.2010	26.1.2010	18.5.2010
Sampling date Chemistry	1.3.2010	10.5.2010	7.12.2009	1.12.2009	18.1.2010	18.1.2010	3.5.2010
Sampling date CH_4_	6.3.2006	3.8.2005			18.3.2003	13.5.2003	4.4.2005
Temperature (°C)	19.6	11.6	17.6	11	14.8	12	17.7
pH	7.9	7.9	7.5	8.1	8.6	7.8	7.3
Ec (mS m^−1^)	897	1832	2190	2670	4110	3770	7820
DIC (mgC L^−1^)	27	4.1	3.9	<3	<3.75	<3.75	<21
NPOC (mgC L^−1^)	10	<2.4	5.1	<3	11	5	<12
TDS (mg L^−1^)	4994	10655	12733	15899	25459	23261	53205
Alk (m) meq L^−1^	2.19	0.37	0.28	0.16	0.29	0.23	0.13
SO_4_^2−^ (mg L^−1^)	79.5	379	2.9	1.4	0.5	0.5	<2
S^2−^ (mg L^−1^)	5.10	NA	0.62	0.02	<0.02	0.13	<0.02
NO_3_ (mg L^−1^)	<0.01	<0.01	<0.01	<0.01	<0.01	<0.01	<0.01
NH_4_ (mg L^−1^)	0.07	0.03	<0.02	<0.02	<0.02	0.04	0.08
Fe^2+^ (mg L^−1^)	<0.02	NA	0.08	0.53	<0.02	0.40	0.46
Na^2+^ (mg L^−1^)	1320	2800	2530	3110	4980	4720	9150
K^+^ (mg L^−1^)	8.2	9.3	8.3	9.6	19	20	27
Ca^2+^ (mg L^−1^)	460	1100	2100	2700	4600	3700	10000
Mg^2+^ (mg L^−1^)	35	77	55	19	18	52	136
Cl^−^ (mg L^−1^)	2920	6230	7930	9940	15700	14600	33500
CH_4_ (mL L^−1^)	22	22	NA	NA	386	272	920
TNC (mL^−1^)	4.2 × 10^5^	1.0 × 10^5^	2.5 × 10^5^	1.5 × 10^4^	5.9 × 10^4^	8.7 × 10^4^	2.3 × 10^4^
*dsr*B gene copies mL^−1^^∗^	3.1 × 10^4^ (8.6 × 10^3^)	5.4 × 10^3^ (2.2 × 10^3^)	1.4 × 10^4^ (6.8 × 10^3^)	1.6 × 10^4^ (9.9 × 10^3^)	6.5 × 10^1^ (2.0 × 10^1^)	2.2 × 10^3^ (2.9 × 10^2^)	0
*dsr*B transcripts mL^−1^^∗^	1.4 × 10^2^ (1.5 × 10^2^)	1.2 × 10^2^ (7.0 × 10^1^)	2.9 × 10^2^ (1.8 × 10^2^)	3.7 × 10^0^ (1.6 × 10^0^)	0	2.0 × 10^1^ (9.0 × 10^0^)	0
*mcr*A copies mL^−1^^∗^	7.5 × 10^0^ (2.5 × 10^0^)	0	5.4 × 10^1^ (2.7 × 10^1^)	0	4.6 × 10^2^ (5.2 × 10^0^)	2.5 × 10^1^ (4.8 × 10^0^)	0
*mcr*A transcripts mL^−1^^∗^	0	0	0	0	0	0	0

NA: data not available.

^*^Figure in brackets shows standard error of mean (SEM).

**Table 2:** Geochemical analysis methods and the detection limit of each assay used in this study. The data were obtained from Posiva Oy.

Parameter	Unit	Method	Detection limit
pH		pH meter, ISO-10532	
EC	(mS m^−1^)	Conductivity analyzer, SFS-EN-27888	5
NPOC	(mg L^−1^)	SFS-EN 1484	TC: 0.6 IC: 0.3 l TOC: 0.3
TDS	(mg L^−1^)		
Alk	(meq L^−1^)	Titration with HCl	0.05
SO_4_^2−^	(mg L^−1^)	IC, conductivity detector	0.1
S^2−^	(mg L^−1^)	Spectrophotometry	0.1
NO_3_^−^	(mg L^−1^)	FIA method, SFS-EN ISO11905-1	0.05
NH_4_^+^	(mg L^−1^)	Spectrophotometry, SFS 3032	
Fe^2+^	(mg L^−1^)	Spectrophotometry	0.01
Na^2+^	(mg L^−1^)	2007: FAAS, SFS3017, 3044 2008: ICP-OES	5 0.5
K^+^	(mg L^−1^)	2007: FAAS, SFS3017, 3044 2008: ICP-OES	0.31 0.5
Ca^2+^	(mg L^−1^)	2007: FAAS, SFS3017, 3044 2008: ICP-OES	0.02 0.1
Mg^2+^	(mg L^−1^)	2007: FAAS, SFS3018 2008: ICP-OES	0.15 0.02
Cl^−^	(mg L^−1^)	Titration	5
CH_4_	(mL L^−1^ gas)	Gas chromatography	1 *µ*L L^−1^ gas

**Table 3:** The primers used for amplification of different microbial groups for 454 pyrosequencing. The archaeal 16S rRNA and the *mcr*A gene transcripts were amplified using a nested PCR approach.

Target	Primer	Sequence	Fragment length (gene location)	Reference
Bacteria 16S rRNA	8F^*^ P2^*^	5′-AGAGTTTGATCCTGGCTCAG-3′ 5′-ATTACCGCGGCTGCTGG-3′	ca. 500 bp (V1–V3)	[23] [24]

Archaea 16S rRNA	A109f Arch915R	5′-ACKGCTCAGTAACACGT-3′ 5′-GTGCTCCCCCGCCAATTCCT-3′	ca. 800 bp	[25] [26]
	ARC344f^*^ Ar744r^*^	5′-ACGGGGCGCAGCAGGCGCGA-3′ 5′-CCCGGGTATCTAATCC-3′	ca. 430 bp (V3-V4)	[27] modified from [28]

Methanogens *mcr*A	*mcr*A412f *mcr*1615r	5′-GAAGTHACHCCNGAAACVATCA-3′ 5′-GGTGDCCNACGTTCATBGC-3′	1.2 kb	[3] [3]
	ME1^*^ ME3r^*^	5′-GCMATGCARATHGGWATGTC-3′ TGTGTGAAWCCKACDCCACC-3′	330 bp	[31] modified from [31]

Sulphate reducer *dsr*B	2060F^*^ dsr4R^*^	5′-CAACATCGTYCAYACCCAGGG-3′ 5′-GTGTAGCAGTTACCGCA-3′	370 bp	[29] [30]

Primers marked with ∗ were equipped with adapter and barcode sequences at the 5′ ends, except if they were used for RT-qPCR. Primers marked with § were used in the qPCR without the adapters and barcodes.

**Table 4:** The number of sequence reads, the observed and estimated number of OTUs, diversity coverage, and diversity index (*H*′) obtained by the HTP sequencing of bacterial and archaeal 16S rRNA and *dsr*B and *mcr*A transcripts. The diversity and OTU richness estimates were calculated based on equal number of sequence reads.

		296 m	328 m	347 m	415 m	559 m	572 m	798 m
Bacteria 16S	Number of reads	1220	893	9209	18425	17158	5377	996
	Observed OTUs	45	46	35	63	83	169	49
	Estimated richness							
	Chao	161	294	73	126	161	367	104
	Ace	412	355	111	219	355	587	139
	Coverage % chao	28	16	48	50	56	46	47
	^∗^ *H*′	1.67	1.25	0.15	0.44	1.79	0.17	1.43

Archaea 16S	Number of reads	6322	12785	1377	2122	1223	1655	3277
	Observed OTUs	139	67	32	45	26	26	6
	Estimated richness							
	Chao	249	137	47	46	46	28	7
	Ace	382	210	81	60	75	30	16
	Coverage % chao	56	49	68	98	57	93	86
	^*^ *H*′	1.06	0.81	1.91	1.23	0.80	1.04	0.83

*dsr*B	Number of reads	8131	4144	12649	8628	2360	2249	—
	Observed OTUs	33	41	47	50	13	26	
	Estimated richness							
	Chao	38	42	51	60	16	31	
	Ace	39	45	52	63	38	49	
	Coverage % chao	86.8	97.6	92.2	83.3	81.3	83.9	
	^*^ *H*′	2.29	2.65	0.69	1.03	1.93	1.81	

*mcr*A	Number of reads	—	4188	4184	—	—	6676	2324
	Observed OTUs		4	2			2	1
	Estimated richness							
	Chao		4	2			2	1
	Ace		5	0			0	0
	Coverage % chao		100	100			100	100
	^*^ *H*′		0.45	0.42			0.76	0.43

^∗^Normalized according to sample with the lowest number of reads.
